# Isolation and identification of a novel goose-origin reovirus GD218 and its pathogenicity experiments

**DOI:** 10.3389/fvets.2024.1423122

**Published:** 2024-10-25

**Authors:** Yuze Li, Huihu Yang, Yongkun Lu, Zhenghao Yin, Hang Xu, Kun Mei, Shujian Huang

**Affiliations:** ^1^School of Animal Science and Technology, Foshan University, Foshan, China; ^2^College of Veterinary Medicine, South China Agricultural University, Guangzhou, China

**Keywords:** novel goose reovirus, separation, whole genome sequencing, genetic analysis, pathogenicity

## Abstract

Since 2020, a novel goose-derived reovirus, characterized by goose hemorrhagic hepatitis, has emerged in the goose breeding industry of Guangdong province, China, leading to significant economic losses in the poultry sector. To study the genetic variation of novel goose reovirus (NGRV) in Guangdong province, this experiment utilized goose embryonic fibroblast cells for virus isolation. RT-PCR was conducted to identify, amplify, clone, and sequence the complete genome of the NGRV isolated from Zhaoqing. The genomic sequences were compared with reference strains to construct a phylogenetic tree. Moreover, animal pathogenicity, excretion patterns, and pathological sections were examined. The results showed that liver and spleen samples from geese suspected of NGRV infection were used for isolation, resulting in the identification of a reovirus presumed to originate from geese, designated as GD218. In terms of genomic structure and sequence homology, GD218 closely resembles the novel duck reovirus, differing significantly from earlier isolated NDRV strains (J18, NP03, SD12, etc.) in genetic composition (nt: 80.6–97.9%, aa: 94.3–98.9%). However, it is similar to strains isolated after 2018, such as XT18, SY, QR, YL, LY20, etc. (nt: 95.3–98.9%, aa: 98.6–99.7%). Therefore, based on phylogenetic analysis, GD218 is hypothesized to be a novel type of goose-origin reovirus homologous to the novel duck reovirus.

## 1 Introduction

In China, avian reovirus is categorized into three types based on its pathogenicity: avian reovirus (ARV), which causes arthritis/tenosynovitis and stunting syndrome; Muscovy duck reovirus (MDRV), responsible for Muscovy duck “liver disease”; and novel duck reovirus (NDRV), which leads to “splenic necrosis” in ducks and geese. Goose reovirus disease mainly affects goslings under 3 weeks old, leading to symptoms such as unstable standing, arthritis, poor growth, neurological manifestations, and pathological changes such as hepatic and splenic necrosis (1, 2). There are two genotypes of goose-origin reovirus: classical goose reovirus (goose reovirus, GRV) and novel goose reovirus (novel goose reovirus, NGRV). The GRV strain was first detected in Guangdong in 2020, predominantly affecting geese under 60 days of age, with necropsy revealing white necrotic foci in the liver and spleen and a mortality rate in flocks reaching up to 20% (3). The NGRV strain was first discovered and reported by Wang Yongkun in 2002 and gradually spread until widespread outbreaks occurred in Guangdong and Shandong provinces by 2020, causing an infectious disease colloquially known as “goose hemorrhagic necrotizing hepatitis” with a variable mortality rate of 15 to 40%, posing a severe threat to the goose farming industry (4, 5).

Goose-origin reovirus belongs to the avian orthoreovirus (ARVs) genus, a double-stranded segmented RNA virus with spherical virions approximately 70–80 nm in diameter, lacking an envelope, and having an icosahedral, double-layered capsid structure (6, 7). The ARV genome contains 10 gene segments (L1, L2, L3, M1, M2, M3, S1, S2, S3, S4) that code for 12 proteins (λA, λB, λC, μA, μB, σA, σB, σC, μNS, P10, P18, σNS), with the S1 gene segment encoding for P10, P18, and σC proteins. The genomic structures of ARV, NDRV, and NGRV are similar, with the S1 gene segment being tricistronic, containing three overlapping open reading frames (ORFs) coding for σC, P10, and P18 proteins. The S4 gene segment of MDRV and GRV is bicistronic, coding for σC and P10 proteins, while the S1 to S3 genes encode for σA, σB, and σNS proteins, respectively (6, 8–11).

In this study, a strain of NGRV was isolated from diseased goose liver and spleen tissue samples collected from Zhaoqing, Shaoguan, Qingyuan, Foshan, and other regions in Guangdong province, named GD218. The strain was isolated and identified, followed by whole-genome analysis, and pathogenicity testing to elucidate its genetic evolutionary patterns and epidemiological status.

## 2 Materials and methods

### 2.1 Sample processing

Appropriate quantities of liver and spleen tissue samples were collected from diseased geese and preserved at the Preventive Veterinary Laboratory of Foshan University of Science and Technology between 2019–2021. They were subsequently minced and mixed with three volumes of sterile PBS (pH = 7.4) containing 1% antibiotic-antimycotic solution (Beyotime, Shanghai, China). The mixture was homogenized, freeze-thawed thrice, centrifuged at 12,000 rpm for 10 min and the supernatant was sterile-filtered and stored for later use.

### 2.2 Virus isolation

The tissue suspension was prepared as described above and centrifuged at 12,000 rpm for 15 min. The supernatant was filtered through a 0.22 μm filter (Jet, Guangdong, China), and mixed with antibiotics to a final concentration of 1% to prepare tissue samples. Once the goose embryonic fibroblast (GEF) cell density reached approximately 80%, the culture medium was discarded, the cells were washed twice with PBS, and 500 μL of the processed tissue samples were inoculated into T25 flask (Thermo Fisher Scientific). After incubation for 1.5 h, the viral fluid was discarded, the cells were washed twice with PBS, and further for 5–7 days in DMEM (Gibco, Grand Island) containing 1% fetal bovine serum. Once a cytopathic effect (CPE) greater than 75% was observed in the cells, the virus culture was collected. The culture was then subjected to three freeze–thaw cycles and passaged until a stable CPE was consistently observed. The viral fluid from each passage was subsequently processed for nucleic acid extraction (Axygen, Hangzhou, China) and virus identification.

### 2.3 Whole genome amplification

Cell viral fluid samples from section 1.2 were amplified using specific primers shown in Table 1. The reaction mixture is presented in Table 2. The amplification program was set at 50°C for 30 min of reverse transcription, 94°C for 3 min of initial denaturation, followed by 35 cycles of 94°C for 30 s of denaturation, 55°C for 30 s of annealing, and 72°C for 10 min of extension. After the reaction, amplified products were run on a 1% agarose gel (Thermo Fisher Scientific, MA), observed, and recorded using a gel documentation system. Sequencing was performed on the amplified products that showed the desired bands.

**Table 1:** Primers used for viral genome sequencing.

Primers	Primer sequences (5′–3′)	Product size (bp)
N-L1a-F	GCTTTTTCTCCGAACGCCGA	2,041
N-L1a-R	TAGGGTCATCCATAGGCAAATTCTC	
N-L1b-F	CCTATGGATGACCCTAACTT	1,934
N-L1b-R	GATGAATAACCTCCAACGA	
N-L2a-F	GCTTTTTCCTCACCATGCAT	1,958
N-L2a-R	TGACACATAACCTGGAAACC	
N-L2b-F	GTCCTCAATGCCTATTTCCG	1,913
N-L2b-R	GATGAGTAATTCCTCGAGCCA	
N-L3a-F	GCTTTTACACCCATGGCTCA	2,118
N-L3a-R	AGTGGGTCGTCCAGCGTAA	
N-L3b-F	CTTTCAATCCCTCCGCTG	1,921
N-L3b-R	GATGAGTAACACCCTTCTACTGGAG	
N-M1-F	GCTTTTCTCGACATGGCCTATCTAGC	2,284
N-M1-R	GATGAATATCTCAAGACGGCTAACCCAGG	
N-M2-F	GCTTTTTGAGTGCTAACCT	2,158
N-M2-R	GATGAGTAACGTGCTAACC	
N-M3-F	GCTTTTTGAGTCCTAGCGTGG	1,996
N-M3-R	GATGAGTAACCGAGTCCGCCGTGG	
N-S1-F	GCTTTTTTCTTCTCTGCCCAT	1,568
N-S1-R	GATGAATAGCTCTTCTCATCGTGC	
N-S2-F	GCTTTTTCTCCCACGATGGC	1,324
N-S2-R	GATGAATACACCCACGCGCTAC	
N-S3-F	GCTTTTTGAGTCCTCAGCGTG	1,202
N-S3-R	GATGAATAGGCGAGTCCCGC	
N-S4-F	GCTTTTTGAGTCCTTGTGCA	1,191
N-S4-R	GATGAATAAGAGTCCAAGTCGC	

**Table 2:** RT-PCR reaction system.

Reagent name	Volume (μL)
PrimeScript^™^ 1 step enzyme mix	1.5
2 × 1-step buffer	15
NGRV-primers-F	1.5 (100 ng/μL)
NGRV-primers-R	1.5 (100 ng/μL)
RNA	3
RNase free H_2_O	Up to 30

### 2.4 Phylogenetic analysis

To reveal the genetic evolutionary relationship between the isolated strains and other avian orthoreoviruses, 26 published avian orthoreovirus strains were selected from GenBank as reference strains, including 13 NDRV, 1 NGRV, 3 MDRV, 2 GRV, and 6 ARV. The sequencing results were compared and analyzed, and a phylogenetic tree was constructed using MEGA 5.0 software through the neighbor-joining method, with bootstrap repetitions set at 1,000 times.

### 2.5 Animal pathogenicity experiments

For the virus pathogenicity experiments, 41-day-old Muscovy ducklings and goslings (from Guangzhou South China Agricultural University Biological Drug Co., Ltd.) were used. The subjects were divided into two groups: 20 ducklings and goslings in the control group and 20 in the infected group. The infected group was injected subcutaneously in the neck with 0.5 mL of virus solution (TCID50 = 10^–4.0^/0.1 mL), and the control group was injected with the same volume of saline. The animals were housed in isolators. Daily observations were conducted for 12 days to monitor animal symptoms and weight changes. Oropharyngeal and cloacal swabs were collected from the infected group at 12 h, and on days 1, 2, 3, 5, 7, 9, and 11 post-infection to analyze excretion patterns. At 1, 3-, 6-, 9-, and 12-days post-infection, four animals from each group were randomly euthanized to examine organ lesions in the infected group. Key organs were collected for tissue section preparation and viral load testing.

### 2.6 Excretion pattern analysis

Oropharyngeal and cloacal swabs were collected, subjected to three freeze–thaw cycles and used for nucleic acids extraction. A SYBR Green real-time fluorescent quantitative PCR method for detecting goose-origin reovirus was established to analyze the samples. Experiments were conducted using TB Green^®^ Premix Ex Taq^™^ II (TaKaRa Biotechnology Company, Dalian, China). The amplification protocol included an initial denaturation at 95°C for 2 min, followed by 40 cycles of denaturation at 95°C for 15 s, annealing at 60°C for 15 s, and extension at 72°C for 30 s. Viral loads in the swabs were calculated from the Cq values to analyze the virus excretion pattern in infected animals’ respiratory and digestive tracts at different time points.

### 2.7 Histopathological observation

During necropsy, liver, spleen, pancreas, and bursa of Fabricius tissues collected at 6 days post-infection (dpi) were fixed in 4% paraformaldehyde (Beyotime, Shanghai, China) for 24 h. The tissue was cut into sections of the designed size.

## 3 Results and analysis

### 3.1 Pathogen isolation results

**Figure 1:**
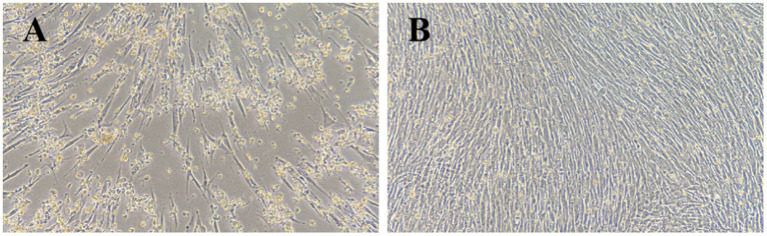
Cytopathic effects in GEF cells 72 h after virus inoculation. **(A)** Test group (100×). **(B)** Control group (100×).

Goose-origin reovirus-positive tissue suspensions were inoculated onto primary GEF cells. Following three consecutive blind passages, the viral fluid induced significant cytopathic effects in the cells within 72 h of inoculation. As illustrated in Figure 1, the cells exhibited rounding, detachment, and extensive necrosis.

### 3.2 Whole genome sequencing and phylogenetic analysis

**Figure 2:**
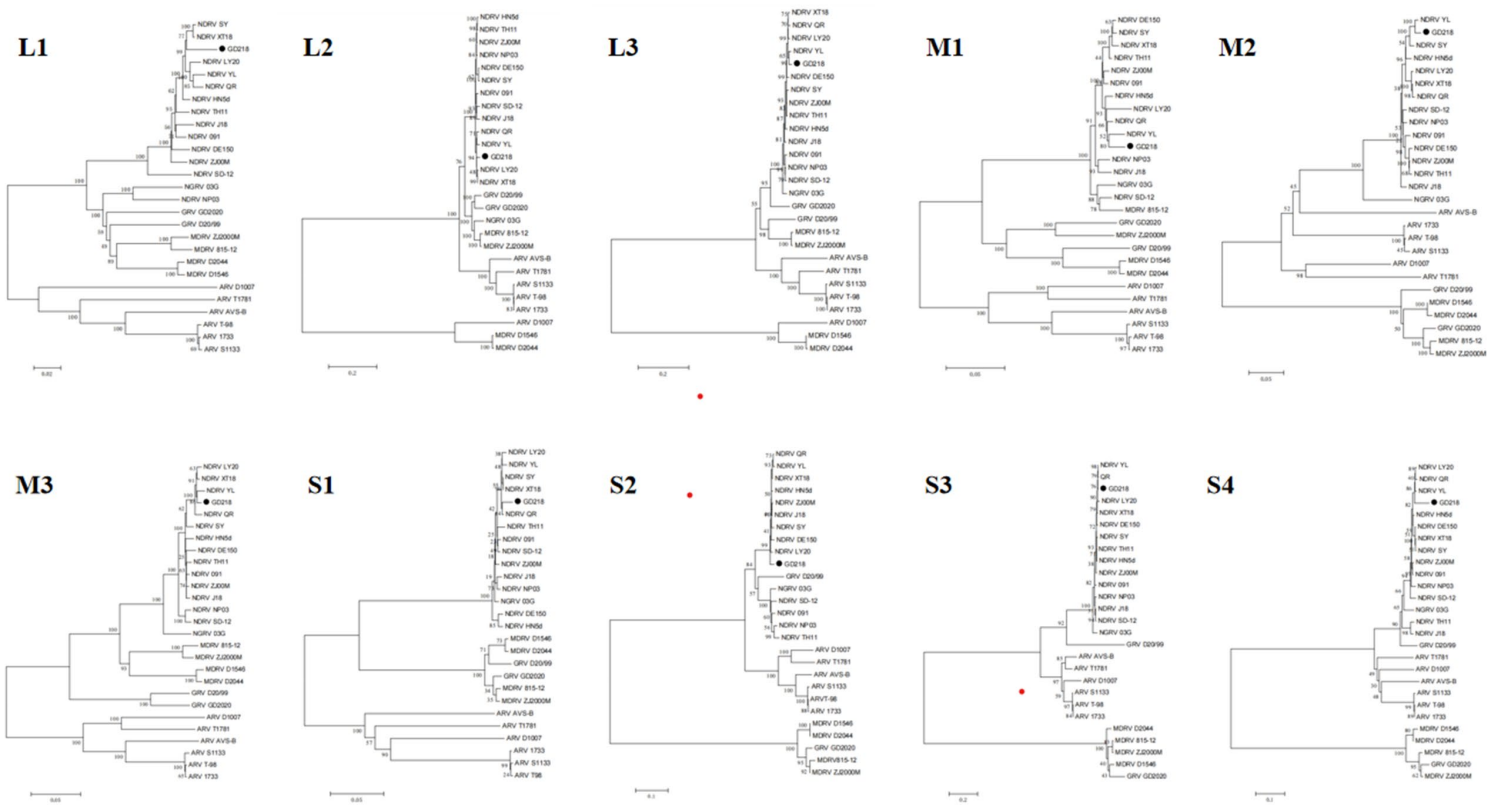
Phylogenetic analysis of the gene of the isolated strain.

The sequencing results were compared and analyzed with the published NDRV, MDRV, GRV, NGRV, and ARV genome sequences in GenBank, and a phylogenetic tree was constructed using MEGA software. Figure 2 shows that the GD218 strain is positioned on the NDRV branch, exhibiting a closer genetic relationship to recently isolated duck-origin reoviruses such as XT18, SY, QR, YL, and LY20. This positioning highlights the genetic distinctions between GD218-purified and other NGRV strains, suggesting that GD218-purified may represent a new variant of waterfowl-origin reovirus derived from the novel duck reovirus.

### 3.3 Pathological results

**Figure 3:**
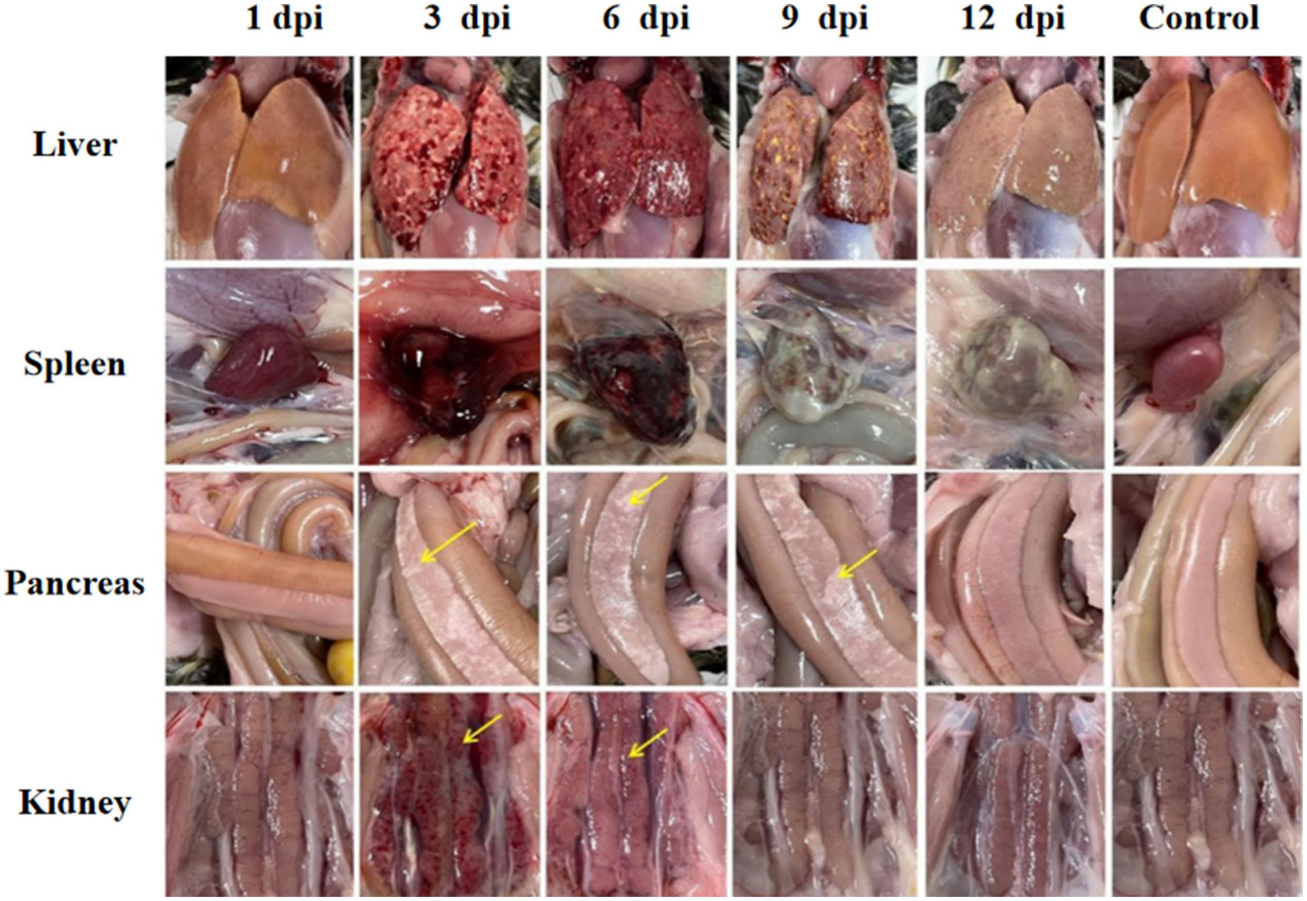
Organ lesion results at different time points after geese were infected with GD218-purified.

**Figure 4:**
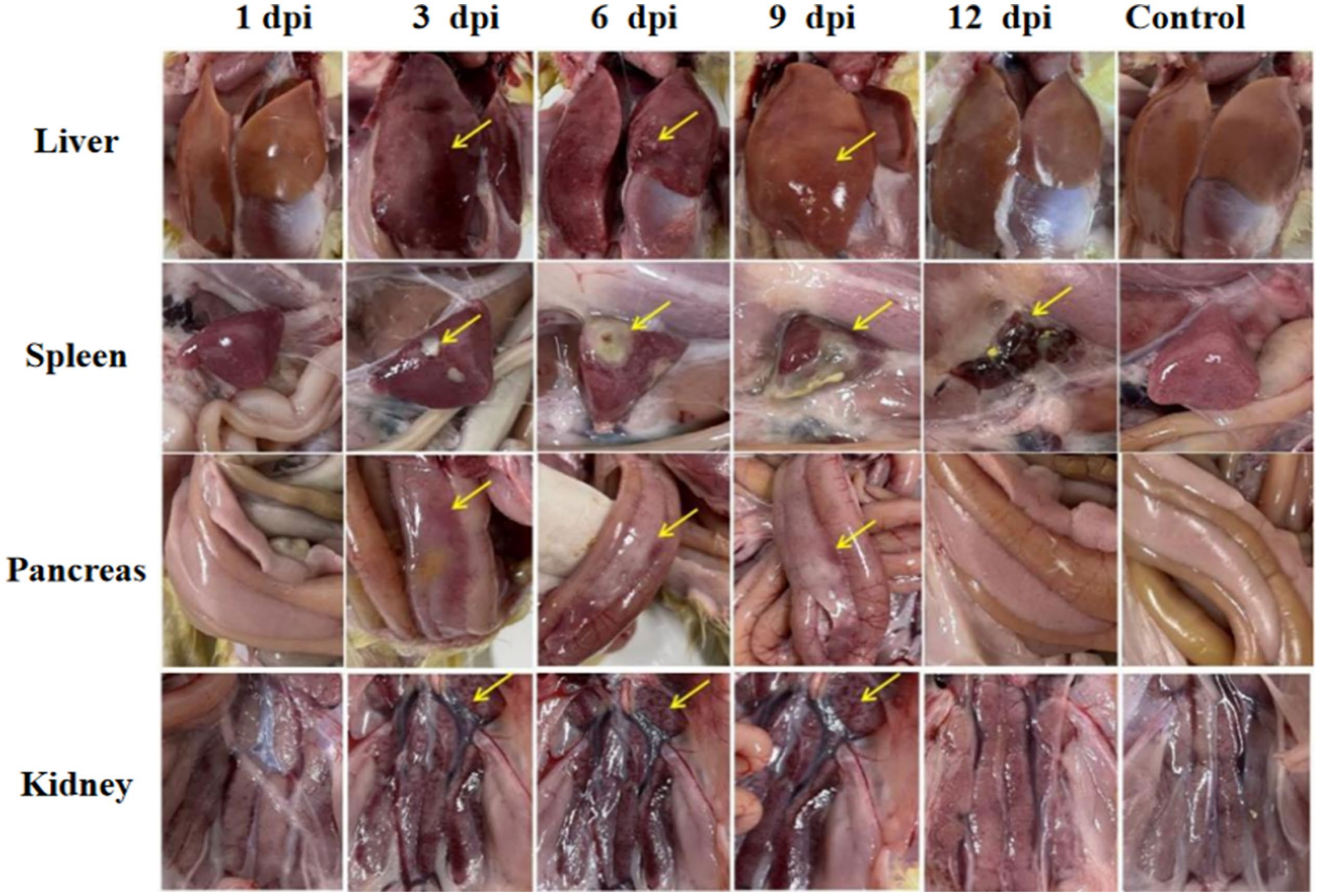
Organ lesion results at different time points after Muscovy ducks were infected with GD218-purified.

Significant visceral lesions were observed in the goose and Muscovy duck groups infected with the GD218-purified strain during necropsy. The extent of lesions varied at different time points; in the goose infection group, no obvious gross lesions were observed at 1 dpi, while at 3 dpi and 6 dpi, severe hemorrhaging and swelling of the liver and spleen were visible, with large areas of white necrosis on the liver’s surface and its texture becoming brittle (Figure 3), pancreatic necrotic foci, and swollen kidney hemorrhage. By 9 dpi, the liver exhibited widespread yellow necrotic foci and dark red hemorrhagic spots, along with severe necrosis, spleen necrosis and atrophy, and pancreatic necrosis. No significant lesions were observed in the kidneys. By 12 dpi, only a few necrotic foci remained in the liver, with no hemorrhage, and the spleen showed signs of necrosis and atrophy. There were no significant lesions in the pancreas or kidneys. In the Muscovy duck infection group, no obvious gross lesions were observed at 1 dpi, while at 3 dpi and 6 dpi, hemorrhaging and swelling of the liver with a few white necrotic points (Figure 4), significant white necrotic foci in the spleen, and hemorrhaging and swelling of the pancreas and kidneys were visible. By 9 dpi, localized necrotic points in the liver were observed without bleeding, spleen necrosis and atrophy, and a few hemorrhagic swellings in the pancreas and kidneys; by 12 dpi, no obvious gross lesions in the liver, severe necrosis and atrophy of the spleen, and no significant pancreatic and kidney lesions.

### 3.4 Excretion pattern analysis

**Figure 5:**
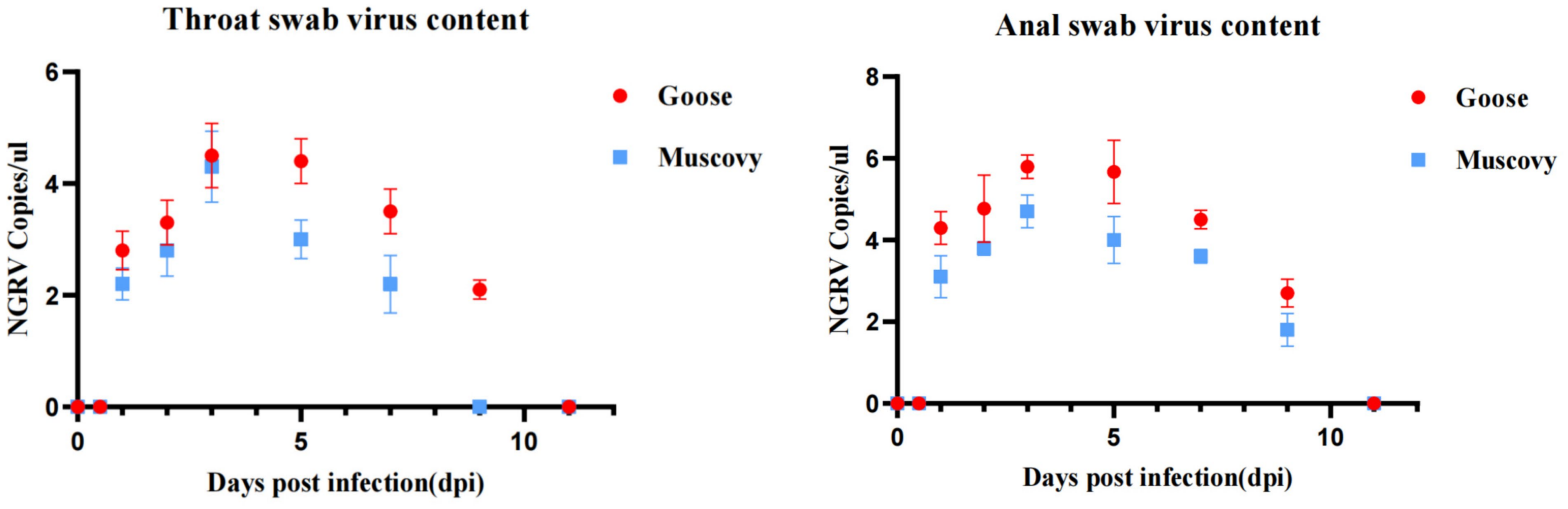
Excretion patterns in the respiratory and digestive tracts after infection with GD218-purified.

Viral presence was detected in oropharyngeal swabs of animals infected with GD218-purified as early as 1 dpi, peaking at 3 dpi (Muscovy ducks: 10^4.3^ copies/μL, geese: 10^4.5^ copies/μL), with goslings showing a longer duration of respiratory excretion. Cloacal swabs began to detect the virus from 1 day dpi, with geese exhibiting significantly higher levels of viral excretion compared to Muscovy ducks. The peak excretion occurred at 3 dpi, with Muscovy ducks showing 10^4.7^ copies/μL and geese showing 10^5.8^ copies/μL. Both infected species maintained elevated levels of viral excretion in the digestive tract from 1 to 7 dpi, with viral copy numbers consistently above 10^–4.4^ copies/μL. Geese had a longer excretion period, with the virus remaining detectable in cloacal swabs until 11 dpi (see Figure 5).

### 3.5 Histopathological results

**Figure 6:**
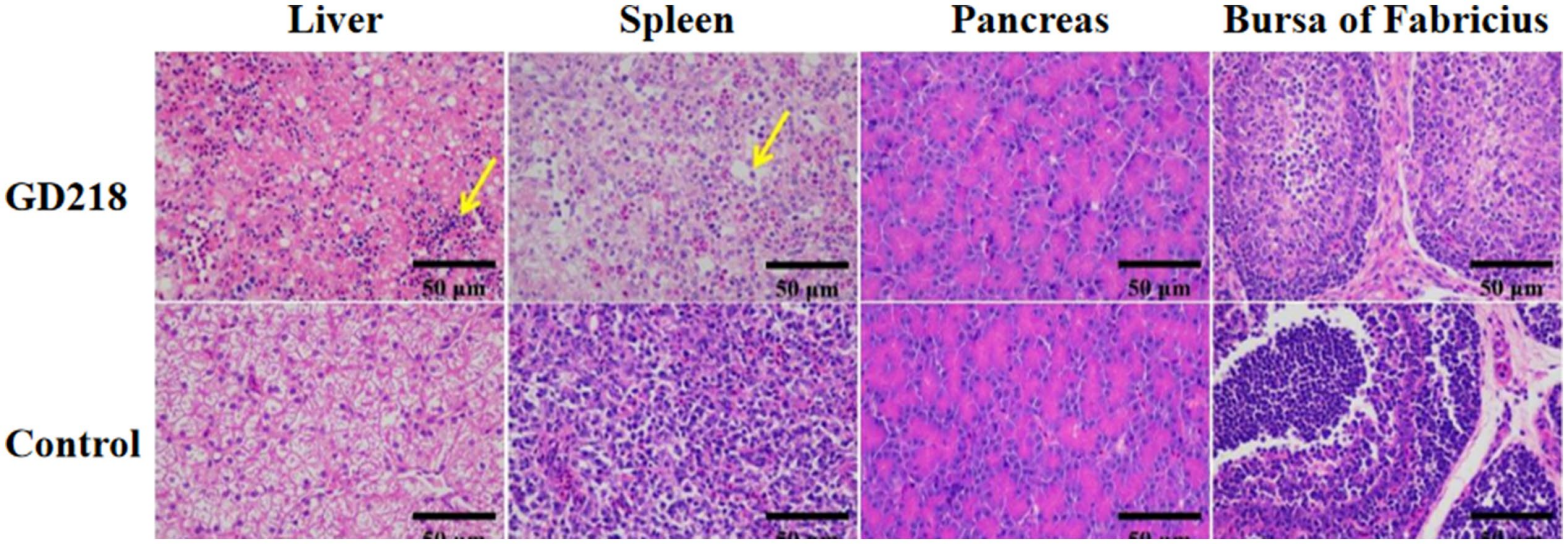
Histopathological examination of organ tissue results at 6 dpi in the GD218-infected goose group.

**Figure 7:**
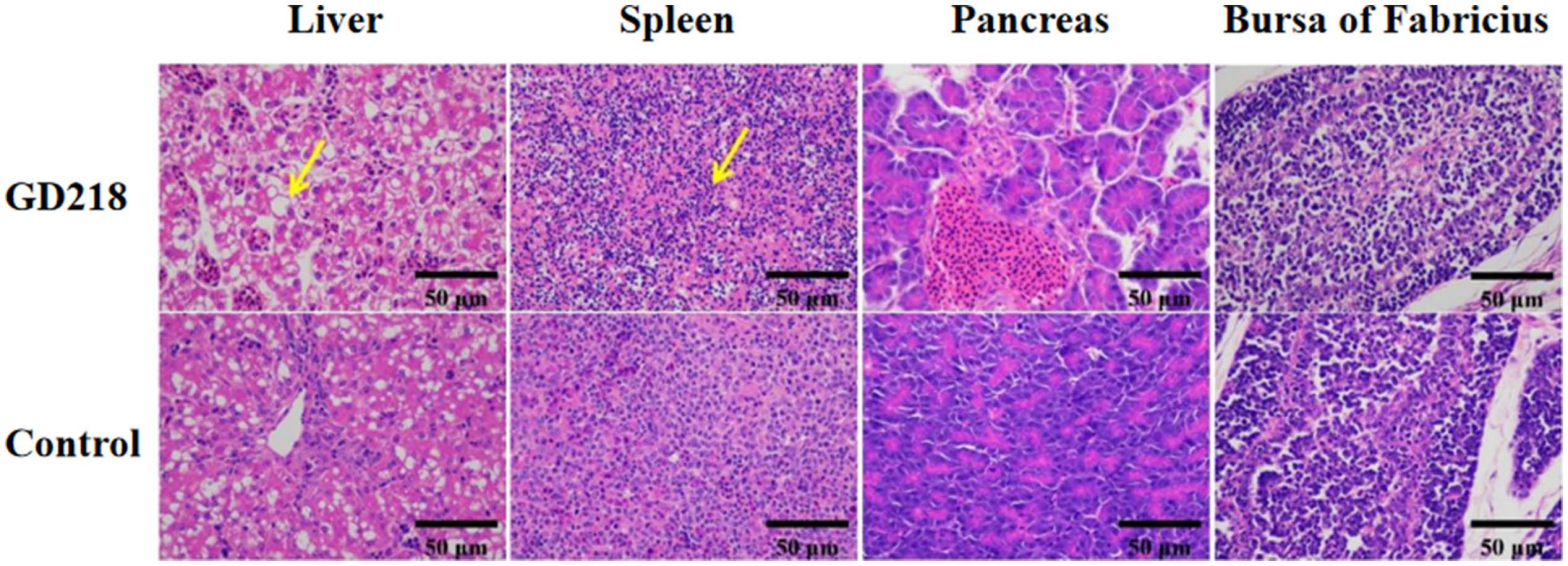
Histopathological examination of organ tissue results at 6 dpi in the GD218-infected Muscovy duck group.

In the GD218-infected goose group (Figure 6), hepatocytes were swollen with nuclei suspended in the cell, diffuse lymphocytic infiltration in the hepatic lobule, shrunken nuclei, sparse organelles, and dilated congested sinusoids filled with red blood cells; severe hemorrhaging in the spleen with widened spleen cell gaps, and shrunken lymphocyte nuclei clustered; no significant gross lesions in the pancreas and bursa of Fabricius. In the infected Muscovy duck group (Figure 7), hepatocytes were swollen, sinusoids were constricted, cytoplasm was highly vacuolated with red-stained granular substances, and localized areas of cell disappearance; spleen lymphocytes were reduced in size, with diffuse shrunken nuclei distributed in the parenchymal spleen cell gaps; pancreatic islets were enlarged and filled with red blood cells; no significant lesions in the bursa of Fabricius.

## 4 Discussion

Avian reovirus was initially identified in broiler chickens exhibiting chronic respiratory ailments in 1954, with its presence in China first documented in 1985 (12, 13). Chen et al. (20) isolated the virus in 1991, leading to its subsequent dissemination across various countries and regions globally. In the year 2023, China produced 4.218 million meat ducks and 515 million commercial geese. As a leading province in domestic waterfowl breeding, Guangdong produced nearly 80 million geese in 2023. However, due to variations in large-scale aquaculture techniques across the country, the incidence of waterfowl-related diseases has increased annually, with both the resurgence of old diseases and the emergence of new ones (14–16). In recent years, the impact of reovirus has steadily increased in densely populated waterfowl breeding regions in China. Due to the virus’s strong environmental resistance, it is frequently detected in clinical testing. The infection induces immune suppression in hosts and increases the risk of secondary infections, resulting in significant economic losses for the waterfowl breeding industry (17–19). Currently, the technology for preventing and controlling waterborne diseases in aquaculture is limited, and such diseases remain to be a major challenge in China, necessitating the search for more effective prevention and control measures.

In this study, a virus was isolated from cases of goose liver and spleen hemorrhagic necrosis between 2021 and 2022, named GD218. Strain GD218 is genomically and sequentially close to NDRV but differs from early isolated NDRV strains (J18, NP03, SD12, etc.) (nt: 80.6–97.9%, aa: 94.3–98.9%) and shows higher similarity to strains reported after 2018 like N-DRV-XT18, DE150, QR, and LY20 (nt: 95.3–98.9%, aa: 98.6–99.7%). On the phylogenetic tree, GD218 is positioned within the NDRV branch, indicating a closer genetic relationship and distance to NDRV strains isolated after 2018, suggesting that these strains played an important role in the formation of GD218.

In this study, we performed infection experiments using the GD218-purified strain on one-day-old goslings and Muscovy ducklings, demonstrating that GD218-purified is pathogenic to both species. Goslings began to die on the third day post-infection, exhibiting a short disease course and a high mortality rate of 40%, which exceeds the mortality rates reported for previous waterfowl-origin reoviruses. In 2017, Zhang (21) isolated a novel goose-origin reovirus strain JS-01 from cases of “goose hemorrhagic necrotic hepatitis,” with a mortality rate of only 20% in infected goslings. However, the mortality rate of GD218-purified observed in this study is significantly higher than that of the JS-01 strain, with more severe visceral lesions noted. Infected Muscovy ducklings began to die on the fourth day post-infection, with necropsy revealing hemorrhagic necrosis in the liver and spleen. The mortality rate in Muscovy ducklings was 15%, comparable to the results of pathogenicity experiments with NDRV on Muscovy ducks.

## Data Availability

The data presented in the study are deposited in NCBI repository, accession numbers PQ391343, PQ391344, PQ391345, PQ391346, PQ391347, PQ391348, PQ391349, PQ391350, PQ391351, and PQ391352.
